# Pre-Attentional Effects on Global Precedence Processing in Children with Autism Spectrum Disorder and Those with Typical Development on a Tablet-Based Modified Navon’s Paradigm Task

**DOI:** 10.3390/healthcare11030372

**Published:** 2023-01-28

**Authors:** Yumi Ju, Soyoung Kang, Jin-Wook Chung, Jeh-Kwang Ryu

**Affiliations:** 1 Human Development and Rehabilitation, Graduate School of Education Service Science, Dongguk University, Seoul 04620, Republic of Korea; 2 Institute for Cognitive Science, Seoul National University, Seoul 08826, Republic of Korea; 3 Department of Sports Culture, Dongguk University, Seoul 04620, Republic of Korea; 4 Department of Physical Education, Dongguk University, Seoul 04620, Republic of Korea; 5 Convergence Research Center for Artificial Intelligence, Dongguk University, Seoul 04620, Republic of Korea

**Keywords:** pre-attentional effects, global precedence processing, autism spectrum disorder, typical development, tablet-based, modified Navon’s paradigm task

## Abstract

This study aimed to characterize the pre-attentional effects on global precedence processing in children with autism spectrum disorder (ASD) and those with typical development (TD). A sample of 17 participants, comprising eight children with ASD and nine TD children, were recruited for the study. A tablet-based assessment utilizing a global and local visual processing paradigm task was developed to investigate the participant’s abilities. The task consisted of verbal instructions to locate and touch either a global or local figure, presented in five conditions: neutral, congruent, and incongruent. The percentage of correct answers and reaction time (RT) for each task were measured and analyzed statistically. Results revealed that children with ASD exhibited statistically significant differences in both the percentage of correct scores and RT among various conditions, while TD children displayed differences in RT but not in the percentage of correct answers. These findings suggest that conflicting processes affect both behavioral and cognitive processes in children with ASD, and that cognitive effort is still involved for children with TD, but does not affect behavioral processes. In children with ASD, the RT was the shortest in the congruent (report local figure) condition; in children with TD, the RT was the shortest in the congruent (report global figure) condition. This implies that children with TD exhibit a pre-attentive effect on global precedence processing, while children with ASD do not. These visual-processing-function characteristics may aid in screening for visual perception problems in children with ASD.

## 1. Introduction

The global prevalence of autism spectrum disorder (ASD) is estimated to be approximately 0.6% [1]. However, the prevalence of autism varies greatly based on country, with a reported rate of 1.89% in Korea [2]. The heterogeneous nature of autism symptoms may account for the discrepancies in reported prevalence, depending on the specificity of diagnostic criteria employed. Moreover, the diagnosis of ASD encompasses a wide spectrum, with a range of symptoms characterized by various aspects of the disorder. According to the *Diagnostic and Statistical Manual of Mental Disorders*, 5th edition (DSM-5), children with a broader range of autistic characteristics are classified as having ASD through an expansion of existing diagnostic criteria [3]. It is crucial to understand the behavioral characteristics of ASD at an individual level for effective intervention and goal-setting, rather than solely relying on a diagnosis.

Children with ASD are known to exhibit general developmental delays in cognitive, motor, and social skills [4,5]. Clinically, specific problem behaviors unique to children with ASD are observed, including inefficient visual searching and aberrant visual perception processing [6,7,8]. For instance, children with ASD frequently demonstrate excessive gazing at a single object and have difficulty processing overall, abstract concepts. Instead, they tend to concentrate on specific, detailed figures, even when playing with toys. This is evidenced by their performance on visual tasks involving the recognition of global figures of characters, such as Navon tasks, which appear to be hindered by the local processing of small characters [9]. Children with ASD exhibit more errors and longer reaction times (RTs) in global processing tasks than children with typical development (TD), which can be explained by Frith’s weak central coherence theory [10]. The theory posits that children with ASD struggle to combine individual component figures into a unitary, coherent unit. It should be noted, however, that while this characteristic is a significant trend in group data, at an individual level, the data are highly heterogeneous and some children with ASD are still able to globally process information [7]. Nevertheless, children with ASD demonstrate more whole–partial interference in visual information processing than children with TD [6]. 

An alternate perspective of the theory of visual information processing in children with ASD suggests that the entire gestalt image is processed first, followed by the processing of detailed information to facilitate a single perception [9,11]. This is referred to as the global precedence hypothesis [9,12]. The global precedence effect is primarily observed in children with TD and is rarely seen in children with ASD [7]. However, the response patterns in cases where global and local figures conflict are different. When the task requires reporting local figures, the response time is longer, and errors increase due to interference from the prioritized global processing. This is known as the cost that arises when global processing is suppressed and local figures proceed [11]. On the other hand, when the task requires reporting global figures, the response time becomes shorter, which is known as the pre-attentional effect, which is due to attentional prompts to engage in global processing.

However, some researchers argue that global and local processing are independent and have a reciprocal relationship, based on inconsistent global or local precedence effects [7]. Thus, cognitive function may not be singularly determined by specific diagnostic groups, and attentional shifting may be attributed to deficiencies in the central execution of executive functions. In fact, research on interdimensional-extradimensional shift tasks has shown that children with ASD have more difficulty with attention shifting than those with TD [13]. Therefore, it is crucial to evaluate global or local processing biases from the perspective of attention shifting when studying children with ASD. 

Previous studies have expressed similar ideas. Soriano’s research involved participants being visually instructed to report global or local information for each trial, in what the researchers called the “switching Navon task.” They argued that this approach allowed them to separate the effects of global and local processing, as well as cognitive shifting, on children’s performance on the Navon task [7]. In this study, instead of providing visual instruction, the researchers gave verbal instruction simultaneously with the stimulus, so that attention-switching could occur within a particular trial. This approach was intended to focus on attention-switching between the levels of one hierarchical stimulus, which is the focal interest of the present study. 

The aim of this study was to investigate the effects of attention shifting on global and local processing, specifically when verbal instructions directed the focus onto one of these levels (i.e., global, or local). The results of this study contribute to existing scientific research by providing evidence that children with ASD are capable of performing global processing, but shifting attention from the local level to the global level requires increased cognitive effort.

## 2. Materials and Methods

### 2.1. Participants 

A total of seventeen participants were recruited, comprising eight children diagnosed with ASD and nine children with TD. The participants were sourced through open recruitment and were compensated with a monetary reward. The study was reviewed and approved by the Dong-guk University Ethics Review Committee (DUIRB-202009-04).

### 2.2. Design of Global-Local Visual Processing Task 

**Figure 1:**
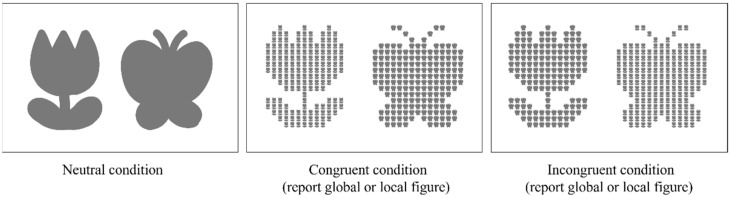
Conditions of the task (Neutral, Congruent (report global figure), Congruent (report local figure), Incongruent (report global figure), Incongruent (report local figure)).

This study utilized a tablet-based modified Navon paradigm task to assess the participants. The task design was based on the Navon paradigm but utilized figures of butterflies and flowers instead of letters for all participants [14]. The task consisted of five conditions, namely form recognition, congruent (report global figure), congruent (report local figure), incongruent (report global figure), and incongruent (report local figure) conditions. The form recognition condition was a neutral condition used to assess the participant’s ability to recognize butterfly and flower shapes before performing the task. In the congruent condition, the global and local figures were the same, whereas, in the incongruent condition, the global and local figures were conflicting (Figure 1). The participants were then verbally instructed to focus on the global or local figures on every single trial, and were required to touch them according to the verbal instruction from the tablet.

The positions of the butterflies and flowers were randomized for each trial. The number of trial repetitions for each condition was 20, resulting in a total of 100 trial repetitions for the participants to perform. The distribution of reports of global and local figures was equally divided in the congruent and incongruent conditions, respectively. The percent correct and RT of each trial were recorded in the data file, with RT being measured as the duration from the onset of the visual stimulus to the touch point.

This study developed a tablet-based task based on the Navon paradigm, for the clinical evaluation of global and local processing efficiency. The task was applied to both groups of children with ASD and TD, and the changes in percent correct and RT in each condition were assessed. Additionally, the potential of the task to differentiate between the two groups of children was evaluated based on the characteristics of the responses under each condition.

### 2.3. Statistical Analysis

Descriptive statistics were used to calculate the correct answer rate and RT in each condition. The non-parametric Friedman test was employed to compare each condition within the groups of children with ASD and TD. A non-parametric Mann−Whitney *U* test was used to analyze the statistical difference between the two groups of children. The statistical analysis was conducted using Jamovi 2.3.16.

## 3. Results

In this study, a total of 17 participants were recruited, consisting of eight children with ASD and nine children with TD. Among the eight children with ASD, five were boys and three were girls. Among the nine children with TD, six were boys and three were girls. The mean age of children with ASD was 9.12 (±1.55) years, and the mean age of children with TD was 8.67 (±0.70) years. The analysis showed that there was no significant difference in the mean age between the two groups (*p* = 0.579). Of the children with ASD, two displayed severe autistic behavioral characteristics, and six displayed moderate probability of autistic behavioral characteristics (Table 1).

In the neutral condition, the percent correct was 92.50% for children with ASD and 100.00% for children with TD. The percent correct was lowest in the incongruent condition among children with ASD, followed by the congruent and neutral conditions. The analysis revealed statistically significant differences in percent correct among all conditions (χ^2^ = 9.52, *p* = 0.049). In contrast, children with TD showed similar percent correct rates under all conditions, ranging from 91.11 to 100.00, with no statistically significant differences among all conditions (χ^2^ = 7.26, *p* = 0.123) (Table 2).

The results of the study revealed that in the neutral condition, the average RT of children with ASD (1.36 ± 0.76 s) was relatively longer than that of children with TD (0.97 ± 0.25 s). Children with ASD had the longest RT in the incongruent condition, followed by the congruent and neutral conditions. Among the four conditions, excluding the neutral condition, the RT was found to be the shortest when reporting local figures in the congruent condition (1.83 ± 0.77 s) and the longest when reporting local figures in an incongruent condition (2.48 ±1.05 s). The analysis revealed statistically significant differences in the RT among all conditions (χ^2^ = 15.1, *p* = 0.005) (Table 2).

In children with TD, the RT was found to be longer in both congruent and incongruent conditions when compared to the neutral condition. Among the four conditions, excluding the neutral condition, the RT was found to be the shortest when reporting global figures in the congruent condition (1.34 ± 0.36 s) and the longest when reporting local figures in the incongruent condition (1.72 ± 0.76 s). The analysis revealed statistically significant differences in RT among all conditions (χ^2^ = 21.6, *p* < 0.001) (Table 2).

Table 3 presented the results of the pairwise comparisons in the post hoc test. In children with ASD, the congruent (report global figure) (t = 0.752, *p* = 0.458) and congruent (report local figure) (t = 1.826, *p* = 0.079) conditions showed no statistically significant differences in percent correct compared to the neutral condition. However, the incongruent (report global figure) (t = 2.792, *p* = 0.009) and incongruent (report local figure) (t = 2.685, *p* = 0.012) conditions showed statistically significant differences in percent correct compared to the neutral condition. For the RT response, the congruent (report global figure) (t = 1.326, *p* = 0.196) and congruent (report local figure) (t = 1.836, *p* = 0.077) conditions showed no statistically significant differences compared to the neutral condition. However, the incongruent (report global figure) (t = 3.875, *p* < 0.001) and incongruent (report local figure) (t = 4.181, *p* < 0.001) conditions showed statistically significant differences in RT compared to the neutral condition. There was no significant difference in percent correct between reporting local and global figures in the congruent (t = 1.074, *p* = 0.292) and incongruent (t = 0.107, *p* = 0.915) conditions. The results in RT also showed no significant differences (t = 0.51, *p* = 0.614; t = 0.306, *p* = 0.762) (Table 3).

In children with TD, the congruent (report global figure) (t = 2.599, *p* = 0.014) and incongruent (report global figure) (t = 2.287, *p* = 0.029) conditions showed statistically significant differences in percent correct compared to the neutral condition. However, the congruent (report local figure) (t = 1.768, *p* = 0.087) and incongruent (report local figure) (t = 1.664, *p* = 0.106) conditions showed no statistically significant differences in percent correct compared to the neutral condition. In terms of RT, all four conditions showed statistically significant differences compared to the neutral condition (Table 3). There was no significant difference in the percent correct between reporting local and global figures in the congruent condition (t = 0.832, *p* = 0.412) and incongruent condition (t = 0.624, *p* = 0.537). The results in RT also showed no significant differences in congruent condition (t = 1.896, *p* = 0.067) and incongruent condition (t = 0.335, *p* = 0.740) (Table 3).

**Figure 2:**
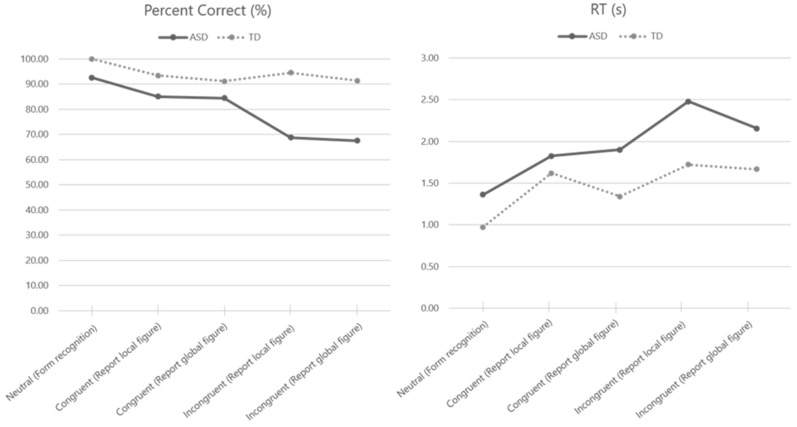
Plot for percent correct (%) and reaction time(s) in each condition among children with ASD and TD.

When comparing the groups of children with ASD and TD, a large difference was observed in the incongruent (report local figure) (MD = −20.00) and incongruent (report global figure) (MD = −10.00) conditions in percent correct (Figure 2), however, none of the conditions between the ASD and TD groups were statistically significant (Table 4). In terms of RT, a large difference was observed in the congruent (report global figure) (MD = 0.618), incongruent (report local figure)(MD = 0.830), and incongruent (report global figure) (MD = 0.628) conditions between children with ASD and TD (Figure 2), however, none of the conditions between the ASD and TD groups were statistically significant (Table 4).

## 4. Discussion

The phenomenon of global and local processing in children with ASD has been described as an integration problem of the processing of local features to the united global figure, as described by the Weak Central Coherence (WCC) theory [10,15]. This hypothesis explains that children with ASD tend to focus on small components rather than perceive the whole picture. However, the reports are heterogeneous and until recently, no agreement has been reached on whether global processing is impaired for children with ASD or other cognitive functions are required for ASD groups to process inputs globally. For example, research by Neufeld et al. supports the WCC theory through their results of a twin study [16], whereas Seerani et al. argue that children with ASD have the ability for global processing but require more time for it [17]. It should be noted that the WCC theory cannot explain all aspects of the phenomena [18].

As opposed to the theory of WCC, which posits that individuals with ASD tend to focus on small components rather than perceiving the whole picture, recent research has suggested that global and local processing may be independent and have a reciprocal relationship based on inconsistent global or local precedence effects [7,12]. Furthermore, studies have found that when provided with global processing instructions, children with ASD perform better than in cases where no such instructions are provided [11]. Although children with ASD may preferentially process local features rather than the global figure, it has been suggested that switching from local to global processing is still possible for children with ASD, provided that attention is directed towards switching between the two processes [19] Soriano et al. have argued that it would be more reasonable to view deficits in global processing in individuals with ASD as a problem of switching from local processing to global processing [7]. The ability to control the switch between the two systems is considered a feature of executive control [20], and research has demonstrated that individuals with ASD may have difficulties in cognitive shifting compared to typically developing individuals in the intradimensional-extradimensional shift paradigm [13]. Ultimately, it is suggested that the switching problem from local to global processing is related to the capacity of the brain to regulate this process [21].

In this study, it was found that children with ASD demonstrated a significant decrease in accuracy in the incongruent conditions, as compared to the congruent conditions (χ² = 9.52, p = 0.049). This indicates that children with ASD exhibited a pronounced compatibility effect resulting from a mismatch in information processing during the task. This contrasts with the results observed in children with TD, who did not display any significant differences in accuracy among the different conditions (χ² = 7.26, p = 0.123), suggesting a lack of a clear compatibility effect in this group. However, it should be noted that both children with ASD (χ² = 15.1, *p* = 0.005) and TD (χ^2^ = 21.6, *p* < 0.001) showed statistically significant differences in RT among each condition. According to Van der Hallen et al.’s meta-analysis, age, gender, and IQ do not affect the performance of global and local processing. Children with ASD can process global information as well as TD children, but children with ASD need more time than TD to process global information with inhibiting local information [8]. In children with ASD, RT was longer in the congruent condition than in the neutral condition, and RT was longer in the incongruent condition than in the congruent condition, i.e., the compatibility effect was also observed in the RT for children with ASD. In children with TD, there was no difference in the percent correct among all conditions, but there was a significant difference in RT. Specifically, although the performance correct rate did not decrease according to the compatibility of the stimulus, RT showed a slowing pattern in children with TD similar to that in children with ASD. That is, in children with TD, if the global and local stimuli conflict, the RT slows down, but the correct answer rates remain stable by engaging cognitive resources. However, it appears that this cannot be overcome due to the inefficiency in processing in children with ASD.

Among the four congruent and incongruent conditions, children with TD had a particularly rapid RT in the congruent (report global figure) condition. In other words, when global and local processing is separated, the global figure is processed first [14], and by giving verbal instruction to find the global figure in the process, global processing is further facilitated. This verbal instruction could operate as a pre-attentive cue to the global level and yield global precedence [22]. This is called the facilitation effect, wherein attention is given to global processing that takes place earlier, and decisions and reactions can be quicker [7].

Further, no pre-attention effect was observed in children with ASD under the congruent (report global figure) condition. Rather, it was observed that RT was the shortest when attention was given to local processing in the congruent (report local figure) condition. That is, children with ASD did not show the same facilitation effect on the global precedence effect, which could be due to global processing not being firm and local preference in children with ASD. Based on previous reports, children with ASD show local figure processing bias and the global precedence effect is weaker than in other diagnostic groups [19,23]. Therefore, an interference effect was expected when verbal instruction was given to focus on the global figure than on the local figure.

The experimental design of this study allowed manipulation of attentional focus by giving verbal instruction to global or local processing. By observing the pre-attentional effect on RT, it is possible to know which processing takes precedence. According to Posner et al.’s attention disengagement theory, it is costly to shift attention to another place by withdrawing attention from the place where the attention resource is applied [24]. Therefore, showing the facilitation effect on global processing when a pre-attentional cue is given supports the global precedence effect, and in contrast, showing the interference effect on global processing demonstrates that local processing is preceded. The results of this study suggest that the global precedence effect does not appear in children with ASD, but rather has a local precedence effect.

The results of this study indicate that in contrast to the congruent condition, the pre-attentional effect was not observed in the global precedence and local precedence effects in the incongruent condition. This may be attributed to the cognitive load evoked by the conflict of incongruent stimuli, which reduces the pre-attentional effect. This is consistent with the findings of Soriano et al. [7], who proposed that when global processing is prompted by an instruction to report a local figure, it induces information inconsistency, which requires the cognitive load to control. During this time, cognitive control encompasses all processes of inhibition and facilitation, and it is the executive function that affects this process of control, not global or local preference. According to the findings of Plaisted et al., selective attention tasks were found to interfere with global precedence processing (interference effect) in children with ASD [11]. However, children with ASD did not exhibit an interference effect when pre-attentional cues were provided verbally in this study. The condition showing the greatest difference between children with ASD and those with TD in correct answer performance was incongruent (report local figure) and incongruent (report global figure) conditions. Although there was no statistically significant difference between the two groups, it is necessary to further verify the statistical significance based on a larger sample size in future studies.

Monitoring conflicts in information processing is an essential cognitive mechanism. Cognitive control, specifically through the anterior cingulate cortex [25], is necessary to regulate these mechanisms, including the monitoring, prevention, and regulation of errors. However, if inhibition is not efficient, it may lead to inadequate regulation of competing processes, resulting in cognitive overload. On the other hand, providing pre-attentional cues to preceding processing has been observed to provide a facilitation advantage. The decrease in the behavioral performance accuracy that appears in the process of conflicting global and local processing can be considered a characteristic of children with ASD. In children with TD, pre-attentive advantage appeared when attention was given by verbal instruction, but children with ASD did not show these characteristics. It implies an inefficient shift between global and local processing and maybe a performance decline due to the difficulty of the inhibitory mechanism in children with ASD. Moreover, knowing the cognitive and perceptual problems of children with ASD provides more useful information in interventions than their diagnosis. By easily evaluating children through tablet-based assessment, it is possible to identify the characteristics of cognitive inefficiency in children with ASD. This study had some limitations. There is a view that the existing Navon paradigm varies depending on duration and size of stimuli, but it has not been considered in this study. However, the tablet-based paradigm in this study can be used simply and practically in clinical situations. In future research, it is necessary to study whether the results of this study show statistical significance in a larger sample group of children. It is necessary to validate whether such cognitive characteristics appear in the ASD group. Further, the results could be supplemented by neuroimaging study while performing the same task, so that the cognitive control in the brain could be confirmed.

## 5. Conclusions

This study aimed to investigate the differences in global and local processing in children with ASD using a tablet-based modification of the Navon paradigm task. The results showed that children with ASD had a significantly lower percent correct in the incongruent conditions compared to the congruent conditions, indicating an obvious compatibility effect due to a mismatch of information processing in the task. In contrast, children with TD did not show a clear compatibility effect. Additionally, the study found that children with TD exhibited a pre-attentional effect in which reaction time was quicker when verbal instructions were given to report the global figure under congruent conditions, but this characteristic was not observed in children with ASD. The study suggests that the global precedence effect is less pronounced in children with ASD than in children with TD, and that this tablet-based assessment may be useful in providing information on cognitive characteristics in intervention.

## Figures and Tables

**Table 1:** Demographic characteristics.

	ASD		TD	Mann–Whitney U
Age (year)	9.12 (±1.55)		8.67 (±0.70)	30.0 (*p* = 0.579)
Gender (n)	M 5/F 3		M 6/F 3	-
Severity of Autism (ABC) ^1^ (n)	Severe	2	-	-
	Moderate	6		

^1^ ABC: Autism Behavioral Checklist, cut off: >68.

**Table 2:** Descriptive statistics of percent correct and response time.

Conditions	Percent Correct (%)				RT (s)			
	ASD		TD		ASD		TD	
	M (SD)	χ^2^	M (SD)	χ^2^	M (SD)	χ^2^	M (SD)	χ^2^
Neutral	92.50 (±17.5)	9.52 (*p* = 0.049)	100.00 (±0.00)	7.26 (*p* = 0.123)	1.36 (±0.76)	15.1 (*p* = 0.005)	0.97 (±0.25)	21.6 (*p* < 0.001)
Congruent (report local figure)	85.00 (±20.0)		93.33 (±11.2)		1.83 (±0.77)		1.62 (±0.66)	
Congruent (report global figure)	86.30 (±18.5)		91.11 (±7.82)		1.90 (±0.63)		1.34 (±0.36)	
Incongruent (report local figure)	68.75 (±32.3)		94.44 (±8.82)		2.48 (±1.05)		1.72 (±0.76)	
Incongruent (report global figure)	67.50 (±36.5)		91.25 (±16.9)		2.16 (±0.58)		1.67 (±0.67)	

**Table 3:** Pairwise comparisons of post hoc (Durbin-Conover).

	Percent Correct (%)					RT (s)		
	ASD		TD		ASD		TD	
	t	*p*	t	*p*	t	*p*	t	*p*
Neutral-Con(global)	0.752	0.458	2.599	0.014	1.326	0.196	2.789	0.009
Neutral-Con(local)	1.826	0.079	1.768	0.087	1.836	0.077	4.685	<0.001
Neutral-Incon(global)	2.792	0.009	2.287	0.029	3.875	<0.001	5.912	<0.001
Neutral-Incon(local)	2.685	0.012	1.664	0.106	4.181	<0.001	5.577	<0.001
Con(local)-Con(global)	1.074	0.292	0.832	0.412	0.51	0.614	1.896	0.067
Incon(local)-Incon(global)	0.107	0.915	0.624	0.537	0.306	0.762	0.335	0.740

Con(local): congruent (report local figure), Con(global): congruent (report global figure), Incon(local): incongruent (report local figure), Incon(global): incongruent (report global figure).

**Table 4:** Group differences between children with ASD and TD in each condition.

	Conditions	MD	U	*p*	95% Confidence Interval	
					Lower	Upper
Percent Correct (%)	Neutral	0.000	27.0	0.144	−10	0
	Congruent (report local figure)	−1.12 × 10^−7^	26.0	0.319	−20	2.25 × 10^−5^
	Congruent (report global figure)	−3.14 × 10^−5^	35.0	0.960	−20.0001	10
	Incongruent (report local figure)	−20.000	16.5	0.052	−50.0001	3.85 × 10^−5^
	Incongruent (report global figure)	−10.000	24.5	0.263	−50	10
RT (s)	Neutral	0.180	23.0	0.236	−0.13	0.79
	Congruent (report local figure)	0.145	33.0	0.815	−0.56	1.07
	Congruent (report global figure)	0.618	19.0	0.112	−0.07	1.16
	Incongruent (report local figure)	0.830	20.0	0.139	−0.23	1.7
	Incongruent (report global figure)	0.628	20.5	0.149	−0.18	1.23

MD: Mean Difference.

## Data Availability

The data that support the findings of this study are available on request from the corresponding author, Jeh-Kwang Ryu.
